# Gum arabic modified Fe_3_O_4 _nanoparticles cross linked with collagen for isolation of bacteria

**DOI:** 10.1186/1477-3155-8-30

**Published:** 2010-12-15

**Authors:** Ashwin Murugappan Chockalingam, Heman Kumar Ramiya Ramesh Babu, Raghuraman Chittor, Jai Prakash Tiwari

**Affiliations:** 1 Centre for Education, Central Electrochemical Research Institute, Karaikudi, Tamilnadu-630006, India; 2 Department of Biotechnology, Kamaraj College of Engineering and Technology, Virudhunagar, Tamilnadu - 626001, India; 3 Functional Materials Division, Central Electrochemical Research Institute, Karaikudi, Tamilnadu-630006, India

## Abstract

**Background:**

Multifunctional magnetic nanoparticles are important class of materials in the field of nanobiotechnology, as it is an emerging area of research for material science and molecular biology researchers. One of the various methods to obtain multifunctional nanomaterials, molecular functionalization by attaching organic functional groups to nanomagnetic materials is an important technique. Recently, functionalized magnetic nanoparticles have been demonstrated to be useful in isolation/detection of dangerous pathogens (bacteria/viruses) for human life. Iron (Fe) based material especially FePt is used in the isolation of ultralow concentrations (< 10^2 ^cfu/ml) of bacteria in less time and it has been demonstrated that van-FePt may be used as an alternative fast detection technique with respect to conventional polymerase chain reaction (PCR) method. However, still further improved demonstrations are necessary with interest to biocompatibility and green chemistry. Herein, we report the synthesis of Fe_3_O_4 _nanoparticles by template medication and its application for the detection/isolation of *S. aureus *bacteria.

**Results:**

The reduction of anhydrous Iron chloride (FeCl_3_) in presence of sodium borohydride and water soluble polyelectrolyte (polydiallyldimethyl ammonium chloride, PDADMAC) produces black precipitates. The X-ray diffraction (XRD), XPS and TEM analysis of the precipitates dried at 373 K demonstrated the formation of nanocrystalline Fe_3_O_4_. Moreover, scanning electron microscopy (SEM) showed isolated *staphylococcous aureus *(*S. aureus*) bacteria at ultralow concentrations using collagen coated gum arabic modified iron oxide nanoparticles (CCGAMION).

**Conclusion:**

We are able to synthesize nanocrystalline Fe_3_O_4 _and CCGAMION was able to isolate *S. aureus *bacteria at 8-10 cfu (colony forming units)/ml within ~3 minutes.

## Background

Exploring rapid and economically efficient technique for isolation/detection of bacteria/viruses at ultralow concentration, alternative to well known conventional technique [1,2] is the need of our modern society. In particular, the use of the nanosized magnetic materials such as Fe_3_O_4_, MFe_2_O_4 _(M = Co, Mn) [3] and FePt [4,5], are reported in literature for bacterial isolation/detection, imaging, drug delivery etc [6-16]. Quite recently a protocol [4] has been reported for isolation/capture of bacteria is based on van-FePt nanoparticles. However, the synthesis procedures for these nanomaterials are relatively complex and expensive in comparison to pure iron oxide nanoparticles [17-19]. Due to their unique magnetic properties, low cost synthesis [19] and low toxicity [12,16,20] Fe_3_O_4 _could be widely used in numerous applications such as cellular labeling [11], magnetic separation [10], tissue repair [21], hyperthermia [21,22], magnetic resonance imaging [7], magnetically guided drug delivery [23] and molecular diagnostics [13] etc. The technique based on super paramagnetic Fe_3_O_4 _nano particles, which respond to an external magnetic field, is an efficient way of separating samples linked to the magnetic particles from the liquid suspension. The particle-linked molecules can quickly agglomerate in the medium in response to a change in external magnetic field. Furthermore, the synthesis reports for iron oxide are based on co-precipitation [24], hydrothermal [15] as well as via high temperature methods [17,25]. However, the wider use of iron oxide based magnetic nanoparticles in biomedical research is still impeded due to the use of toxic chemicals [26], low yield, problems in achieving small, uniform and highly dispersed nano particles. So the desired method of synthesis needs a simpler, economical as well as a low temperature process for their enhanced applications in isolation/detection of dangerous bacteria for humanity. Henceforth, we present a high yield, room temperature, one pot and water based new synthetic protocol that yields iron oxide nanoparticles. Moreover, we have demonstrated instant detection/isolation of pathogenic bacteria *Staphylococcus aureus (S. aureus*) at ultralow concentrations using CCGAMION synthesized through this new protocol, achieving a detection limit of 8 cfu/ml in 3 minutes. *S. aureus *is a gram-positive, perfectly spherical bacterium about 1 μm in diameter. This bacteria causes skin lesions such as boils, styes, furuncles, pneumonia, mastitis, phlebitis, meningitis and urinary tract infections etc. *S. aureus *shows affinity for a wide range of mammalian plasma and extracellular matrix proteins. Among the proteins, collagen was estimated to bind with receptor present on *S. aureus *[27]. Collagen is the main protein of connective tissues and most of the pathogenic bacteria are attached with collagen for colonization and seems to be better choice with respect to antibiotic vancomycin [4]. Further, Iron oxide particles are bio-compatible and suitable for functionalization with gum arabic (GA), a natural polymer which is known for its usage in controlled drug delivery systems and is also a surface active molecule capable of improving magnetic nano particle stability in aqueous solutions by providing steric stabilization [28]. In nutshell, this report is the first kind of demonstration via Fe_3_O_4 _with GA as well as collagen.

## Results

### Characterization of Fe_3_O_4 _nano particles

**Figure 1:**
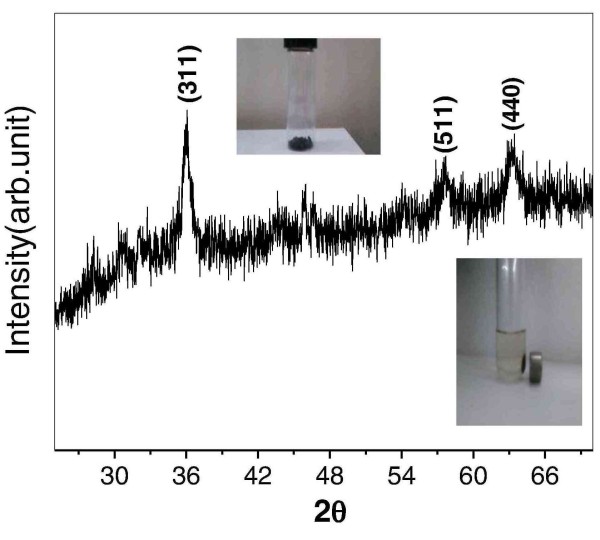
**X-ray diffraction and magnetic nature of material**. The XRD pattern of as-synthesized Fe_3_O_4 _nanoparticles clearly demonstrates the formation of nanocrystalline Fe_3_O_4_. The inset (upper) of figure shows the appearance and color of as-synthesized powder. The other inset (lower one) of the figure shows photograph of drawn nano particles to the sidewall of the vial by an external magnet, dispersed in water.

**Figure 2:**
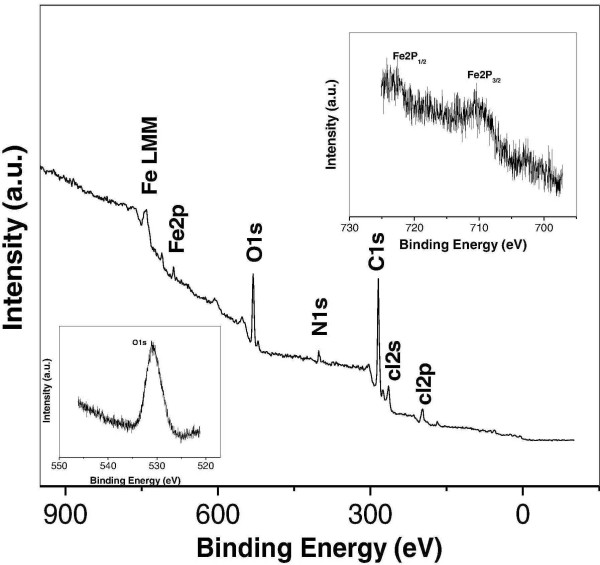
**X-ray photoelectron spectroscopic analysis**. In order to depict the various oxidation states present in the as synthesized iron oxide nanoparticles the XPS spectrum is shown. The inset of this figure shows binding energies of oxygen as well as that of the iron.

**Figure 3:**
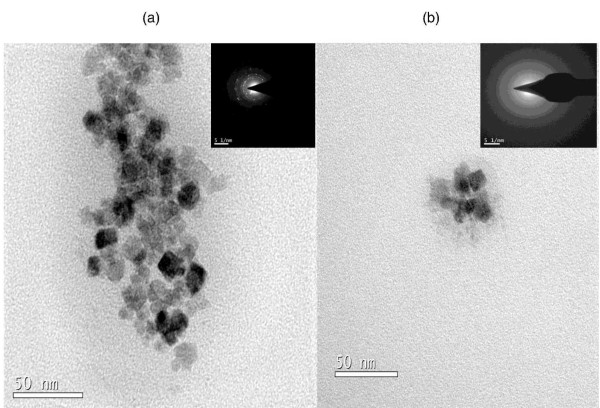
**Transmission electron micrographs (TEM)**. Electron microscopy was done on the as-synthesized iron oxide showing (a)-(b) morphology as well as (inset of Figure 3a-b) respective selected area electron diffraction pattern of the fine grained nanosized Fe_3_O_4 _particles.

The X-ray diffraction data (Figure 1) corresponds to the formation of magnetite (Fe_3_O_4_) nanocrystals [18]. All the peaks of Figure 1 can be indexed to Fe_3_O_4 _structure (JCPDS-88-0315). One can easily observe the broadening of peaks of Fe_3_O_4 _due to crystallite size reduction. The crystallite size calculated using Debye Scherer formula [29] is found to be ~11 nm. The inset (upper one) of Figure 1 shows obvious black appearance of as-synthesized powder. The other inset (lower one) of Figure 1 shows that the nanoparticles dispersed in water can be drawn by applying an external magnetic field. Also, due to XRD pattern similarities of Fe_3_O_4 _and Fe_2_O_3_, the survey XPS spectrum together with spectra of Fe (2p_3/2_,_1/2_) (inset of Figure 2) and O(1s) (inset of Figure 2) is collected and shown in Figure 2. The measured peaks are Fe (1s^2^, 2s^2^, 2p^6^, 3s^2^, 3p^6^, 3d^6^, and 4s^2^), oxygen (1s), nitrogen (1s), carbon (1s) and chlorine (2 s, 2p). The peak at ~715 eV corresponds to Fe2P_3/2 _of Fe^3+ ^and a small peak at ~723 eV corresponds to Fe2P_1/2 _confirming formation of magnetite [30,31]. In addition, the peaks corresponding to sodium, nitrogen, chlorine and carbon originate from PDADMAC indicating the existence of PDADMAC on the surface of iron oxide. Moreover, morphology as well as size of thus-obtained magnetic nanoparticles was investigated through transmission electron microscopy and represented in Figure 3. As it's obvious from Figure 3 the particle size is ~20 nm with non spherical morphology, which is again supported by our observation of crystallite size through XRD peak broadening (Figure 1).

### Bacterial Isolation

**Figure 4:**
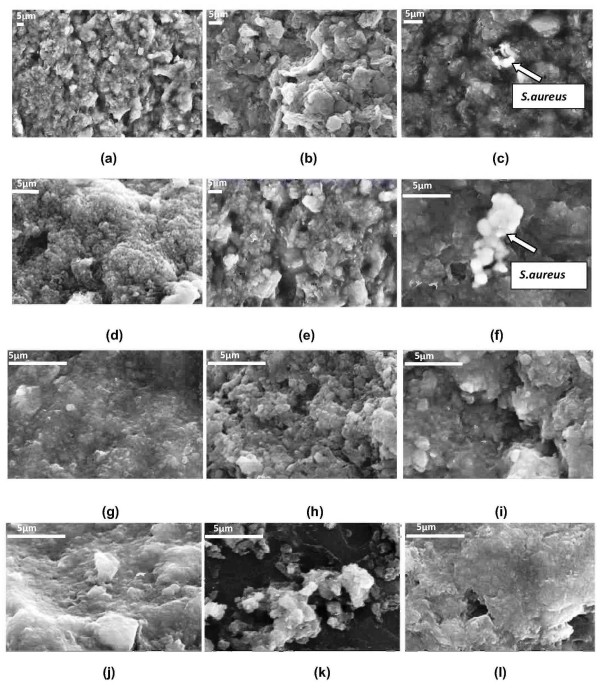
**Scanning electron micrographs (SEM) of various bacterial isolation trials**. The trials were performed using 200 μl PBS, 200 μl water, 200 μl collagen and 30 mg Fe_3_O_4_. (a) Fe_3_O_4 _nanoparticles separated from a solution of 500 μl bacterial (*S. aureus*) solution (~8-10 cfu/ml) + PBS + water + bare Fe_3_O_4_. (b) GA modified Fe_3_O_4 _nanoparticle*s *from a solution of 500 μl bacterial (*S. aureus*) solution (~8-10 cfu/ml) + PBS + water + GA modified Fe_3_O_4. _(c) Aggregates of isolated bacteria (*S. aureus*) and that of the CCGAMION from a solution of 500 μl bacterial (*S. aureus*) solution (~8-10 cfu/ml) + PBS + water + collagen + GA modified Fe_3_O_4_. (d) Fe_3_O_4 _nanoparticles from a solution of 500 μl bacterial (*S. aureus*) solution (~30-40 cfu/ml) + PBS + water + bare Fe_3_O_4_. (e) GA modified Fe_3_O_4 _nanoparticles from a solution of 500 μl bacterial (S. aureus) solution (~30-40 cfu/ml) + PBS + water + GA modified Fe_3_O_4_. (f) Aggregates of isolated bacteria (*S. aureus*) and that of the CCGAMION from a solution of 500 μl bacterial (*S. aureus*) solution (~30-40 cfu/ml) + PBS *+ *water + collagen + GA modified Fe_3_O_4_. (g) Fe_3_O_4 _nano particles from a solution of 500 μl bacterial (*S. albus*) solution (~30-40 cfu/ml) + PBS *+ *water + bare Fe_3_O_4_. (h) GA modified Fe_3_O_4 _nanoparticles from a solution of 500 μl bacterial (*S. albus*) solution (~30-40 cfu/ml) + PBS + water + GA modified Fe_3_O_4_. (i) CCGAMION from a solution of 500 μl bacterial (*S. albus*) solution (~30-40 cfu/ml) *+ *PBS + water + collagen + GA modified Fe_3_O_4_. (j) Fe_3_O_4 _nanoparticles from a solution of 500 μl bacterial (*E. coli*) solution (~30-40 cfu/ml) + PBS + water + bare Fe_3_O_4_. (k) GA modified Fe_3_O_4 _nanoparticles from a solution of 500 μl bacterial (*E. coli*) solution (~30-40 cfu/ml) *+ *PBS + water *+ *GA modified Fe_3_O_4_. (l) CCGAMION from a solution of 500 μl bacterial (*E. coli*) solution (~30-40 cfu/ml) + PBS + water + collagen + GA modified Fe_3_O_4_.

**Figure 5:**
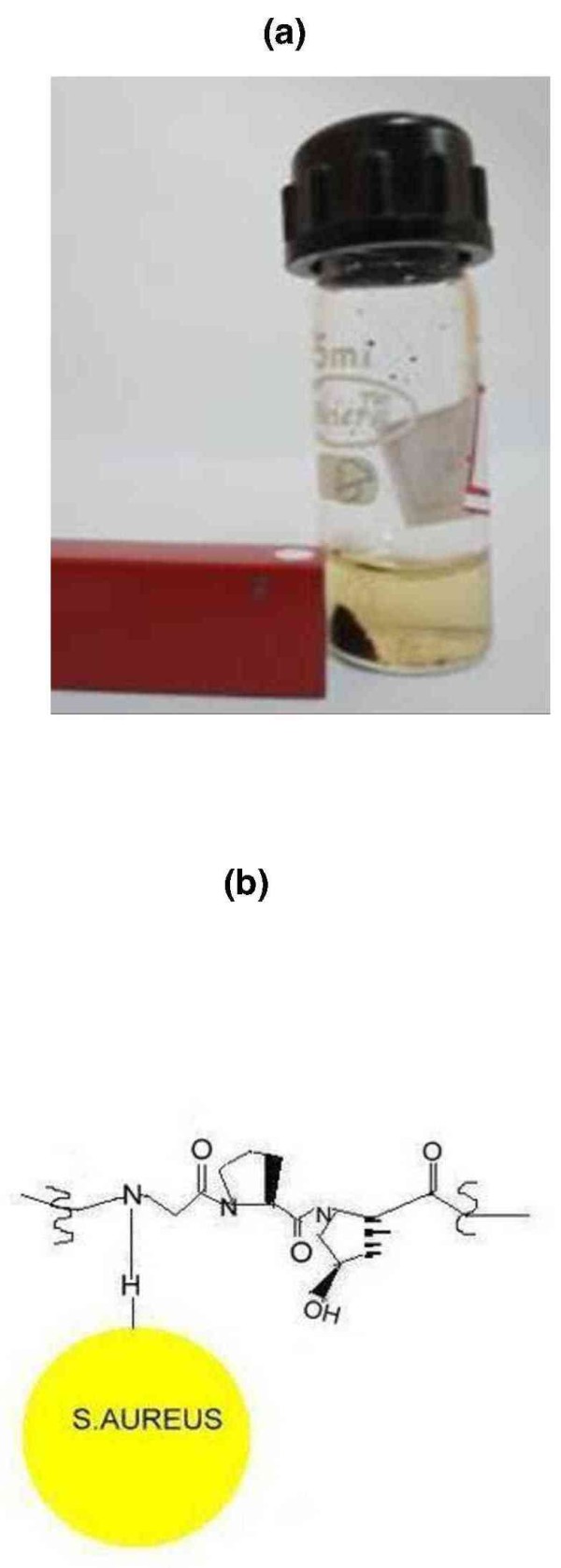
**Demonstration of bacterial isolation process**. (a) The isolation of aggregates of bacteria (*S. aureus*) and CCGAMION from a solution containing 500 μl bacterial (*S. aureus*) solution (~8 cfu/ml) + 200 μl PBS + 200 μl water *+ *160 μl collagen + 30 mg of GA modified Fe_3_O_4 _(b) Cartoon representation of the interaction of bacteria with that of fish collagen.

A representative image of the captured bacteria with the help of CCGAMION at various cfu/ml is shown in Figure 4. This figure clearly shows that CCGAMION can capture only *S. aureus *from 8-40 cfu/ml (Figure 4c and Figure 4f) and it is not able to capture other bacteria such as *S. albus *and *E. coli *(Figure 4i and Figure 4l) which may be due to less affinity of *S*. albus/*E. coli *towards collagen binding sub segment CNA [27]. It is obvious that with the increase of concentration of bacterial solution, we can easily isolate bacteria from the solution. Herein, mixing of gum arabic modified Fe_3_O_4 _with collagen to bacterial solution (Figure 5a) results in sufficient number of magnetic nanoparticles binding onto *S. aureus *due to its affinity towards collagen. A small magnet placed near to these simply attracted (Figure 5a) bacteria-nanoparticle composites for the analysis and scanning electron microscopy (SEM) easily detected the bacteria from aggregates due to its micron size and shape (Figure 4c and Figure 4f).

## Discussion

The reduction of iron salt (FeCl_3_) in the presence of structure directing agent PDADMAC, may have nucleated iron (Fe) nanoparticles in the channel created by electrostatic adsorption of BH^-4 ^on the surface of PDADMAC, followed by subsequent oxidation of iron (Fe) to Fe_3_O_4 _on heating at 100°C. However, the role of surfactants in the formation of directed morphologies is a matter of thorough investigations of nucleation, growth and also of interaction energies of surfactants with that of the embryos [32]. Furthermore, the CCGAMION was separated along with the bacteria using the interactions of magnetic nanoparticle-aggregate with an external magnetic field (Figure 5a) and ligand-receptor with that of the bacteria (Figure 5b).

Herein, it is very important to discuss about the interaction of GA with Fe_3_O_4 _and also with collagen. GA attracts Fe_3_O_4 _via electrostatic attraction between carboxylic group of GA and surface hydroxyl group of Fe_3_O_4 _which is due to the glycoprotein present in gum arabic [33]. Furthermore, it is also well known that the adsorption of GA on to the surface of Fe_3_O_4 _follows the Langmuir isotherm indicating that absorption is likely independent of molecular weight of gum Arabic (GA) [28]. As we know that GA is a mixture of branched polysaccharides and glycoprotein containing numerous functional groups which may be responsible for the cross-linking with collagen via covalent bonding [34]. Even naked magnetic nano particles can be absorbed by some bacteria like *E. coli *and *Salmonella *randomly at much higher concentrations (10^5 ^cfu/ml) [35]. On the other hand, our motive is to make Fe_3_O_4 _nano particles to adhere on the surface of *S. aureus *and this can be done only by coating GA modified nanoparticles with collagen. Furthermore, the collagen is a well known mammalian fibrous proteins found in the connective tissues of mammals for providing structural support for tissues, bones, tendons and skin. *S. aureus *has been shown to bind with specific affinity towards collagen due to microbial surface component recognizing adhesive matrix molecules (MSCRAMM) as they are found on the cell surface of the *S aureus*. The collagen binding MSCRAMM on *S. aureus *is called collagen adhesion (CNA) [27,36] and plays an important role in pathogenesis. CNA has structural characteristic of cell wall anchored proteins on gram-positive bacteria and it consists of an N-terminal signal peptide, a non-repetitive region, one to four repeated units, followed by a cell-wall anchor region, a transmembrane segment and a short positively charged cytoplasm tail. The non-repetitive region of CNA is found to be fully responsible for the collagen-binding activity of CNA. Here, it is worth mentioning the presence (Figure 2) of some of PDADMAC with bare Fe_3_O_4 _particles. However, PDADMAC works as surface modifier for achieving a shaped morphology of Fe_3_O_4_. It does not affect the use of Fe_3_O_4 _in the isolation process of bacteria as we are applying a much thicker and better coating through GA.

Furthermore, high sensitivity, selectivity and affinity of bio-functional Fe_3_O_4 _particles for the detection/isolation of bacteria, also depends on the size of magnetic nanoparticles. The size of Fe_3_O_4 _nanoparticles should be such that it can allow the presence of sufficient number of ligands to achieve a multiple interaction, simultaneously it should also be able to yield high surface to volume ratio, stability as well as high binding rates. Due to the significant size differences between Fe_3_O_4 _(~20 nm, Figure 3) nanoparticles and bacteria (micron size, Figure 4), a scanning electron microscope (SEM) easily distinguishes *S. aureus *from the aggregates. Moreover, the smaller size as well as high surface/volume ratio of Fe_3_O_4 _nanoparticles increases its biding efficiency with bacteria. Besides, the above stated size requirements; Fe_3_O_4 _nano particles have biocompatibility and biodegradability for *in vivo *applications. However, for our *in vitro *application good chemical stability of Fe_3_O_4 _is adequate.

## Conclusions

In summary, we have succeeded in synthesizing Fe_3_O_4 _nanoparticles through a wet chemical route using PDADMAC as capping agent. Reactive Fe nanoparticles initially formed due to redox reaction using PDADMAC as a soft template which subsequently turns into Fe_3_O_4 _nanoparticles on heating at 100°C through oxidation process. Using collagen coated gum arabic modified Fe_3_O_4 _nano particles, we are able to capture, collagen binding bacteria *S. aureus *at various concentration ranging from 8 cfu/ml to 40 cfu/ml. However, we need a deeper study of surface chemistry for attaching bioactive molecules onto a magnetic nanoparticles as well as more precise control of numbers and orientations of molecules. Moreover, the CCGAMION may provide a new technology platform for isolation/detection of *S. aureus *and we can expect a major role of nanosized Fe_3_O_4 _materials in diagnostics and clinical applications in near future.

## Methods

### Materials

The fish collagen (Biofill) was purchased from Eucare pharmaceuticals (origin fish), Chennai (India). The anhydrous ferric chloride was purchased from Central Drug House (India), New Delhi. Gum arabic (GA), nutrient broth and agar-agar were purchased from Himedia, Mumbai (India). PDADMAC from Sigma Aldrich chemicals. *Staphylococcus aureus, staphylococcus *albus, *Escherichia *coli was isolated from patients sample at Bose Clinical Laboratory, Madurai (India).

### Synthesis of Fe_3_O_4_

The anhydrous ferric chloride salt (10 mM) was dissolved in 50 ml of ethanol and allowed to react with a solution containing a mixture of 10 ml PDADMAC, 50 ml of water and NaBH_4 _(100 mM) in ethanol for about one hr with constant magnetic stirring. The final reaction mixture is allowed to stand for one hour for the precipitation. The mixture was centrifuged and precipitate was removed. Further stoving was done at 100°C for 12 hours for late ripening of the powders and used for various characterizations. The X-ray powder diffraction (XRD) was carried out on an X'Pert PRO PANalytical instrument with Cu K_α _radiation at a scanning rate of 2° per min. The morphological pictures of nanoparticles were taken with the help of Technai G^2 ^type of transmission electron microscope (TEM). Moreover, the samples are analyzed with the help of XPS-multilab-2000 (thermoscientific, U.K.).

### Surface modification of Fe_3_O_4 _with GA

~1 g of Fe_3_O_4 _was dispersed in GA solution (5 mg/ml) and sonicated for 15 mins. Then 100 Gauss bar magnet is used to bring down the particles at a corner of the stopper bottle and the particles were dried overnight.

### Preparation of collagen solution

~0.5 g of fish collagen particles were dissolved in 0.5 M acetic acid and sonicated for 10 min. Finally, the mixture is filtered through 5 micron filter paper to get a clear collagen solution.

### Preparation of bacterial solution

Bacterial cells were suspended in peptone water and serial dilutions were made until the desired concentration of ~8 to ~40 cfu/ml was established.

### Procedure

In trial, 30 mg of GA modified Fe_3_O_4 _was dissolved to 5 ml vial containing a mixture of 200 μl PBS, 200 μl water and 160 μl fish collagen with a constant stirring up to 5 minutes. Then immediately after adding 500 μl (10 cfu/ml) of bacterial solution, magnetization was done using a bar magnet of 100 gauss up to 3 minutes, taken as the capture time of bacteria. After 10 min the aqueous solution was carefully removed using a micro pipette, 0.5 ml from each sample is poured on nutrient agar plates and incubated overnight at 37°C.

The aggregates were analyzed with the help of Hitachi model S-3000 H scanning electron microscope. All the materials except bacterial solutions were sterilized with UV-rays before use. Further, similar experiments were repeated with *S. albus *(~30-40 cfu/ml) and *E. coli *(~30-40 cfu/ml) strains. The concentration of bacteria left over in the vial in terms of cfu/ml for all the samples analyzed and tabulated in Table 1.

**Table 1:** The number of colony forming units (cfu) of bacterial samples left in the vial after magnetic isolation.

500 μl bacterial Solution (cfu/ml)	**200 μl PBS+360 μl water + 30 mg bare Fe**_**3**_**O**_**4 **_**(cfu/ml)**	**200 μl PBS+360 μl water + 30 mg GA modified Fe**_**3**_**O**_**4 **_**(cfu/ml)**	**200 μl PBS+200 μl water+30 mg GA modified Fe**_**3**_**O**_**4 **_**+ 160 μl collagen (cfu/ml)**
8-10, *S. aureus*	~8	~8	~2
30-40, *S. aureus*	~32	~30	~6
30-40, *S. albus*	~28	~29	~30
30-40, *E. coli*	~31	~36	~26

## List of abbreviations

GA: Gum Arabic; CCGAMION: Collagen coated gum Arabic modified Iron Oxide Nanoparticles; S. AUREUS: *Staphylococcus aureus*; S. ALBUS: *Staphylococcus *albus; *E. COLI*: *Escherichia coli*; CFU: Colony forming unit; PDADMAC: polyelectrolyte (polydiallyldimethyl ammonium chloride); XRD: X-ray diffraction; XPS: X-ray photo electron spectroscopy; TEM: Transmission electron microscopy; SEM: Scanning electron microscopy; PBS: Phosphate Buffered Saline; PCR: Polymerase chain reaction; VAN-FEPT: Vancomycin- Iron Platinum; MSCRAMM: Microbial surface component recognizing adhesive matrix molecules.

## Competing interests

The authors declare that they have no competing interests.

## Authors' contributions

JPT and AMC co-ordinated experiments and provided important advice for the experiments. AMC, HKRRB and RC performed the majority of the experiments. AMC and JPT performed the majority of characterization. All authors read, participated in writing and approved the final manuscript.

## References

[B1] Che YH Li YB Slavik M Paul D Rapid Detection of Salmonella typhimurium in chicken carcass wash water using an immunoelectrochemical method J food Prot 2000 63 1043 1048 10945578 10.4315/0362-028x-63.8.1043

[B2] Wagner SJ Robinette D Evaluation of an automated microbiologic blood culture device for detection of bacteria in platelet components Transfusion 1998 38 674 679 10.1046/j.1537-2995.1998.38798346637.x 9683107

[B3] Robinson DB Persson HHJ Zeng H Li GX Pourmand N Sun SH Wang SX DNA-Functionalized MFe_2_O_4 _(M = Fe, Co, or Mn) Nanoparticles and Their Hybridization to DNA-Functionalized Surfaces Langmuir 2005 21 3096 3103 10.1021/la047206o 15779990 PMC2924586

[B4] Gu H Xu K Xu C Xu B Biofunctional magnetic nanoparticles for protein separation and pathogen detection Chem Comm 2006 9 941 949 10.1039/b514130c 16491171

[B5] Gu H Ho PL Tsang KWT Wang L Xu B Using Biofunctional Magnetic Nanoparticles to Capture Vancomycin-Resistant Enterococci and Other Gram-Positive Bacteria at Ultralow Concentration J Am Chem Soc 2003 125 15702 15703 10.1021/ja0359310 14677934

[B6] Perez JM Iron oxide nanoparticles: Hidden talent Nat Nanotechnology 2007 2 535 536 10.1038/nnano.2007.282 18654361

[B7] Gao J Gu H Xu B Multifunctional Magnetic Nanoparticles: Design, Synthesis, and Biomedical Applications Acc Chem Res 2009 42 1097 1107 10.1021/ar9000026 19476332

[B8] Fornara A Johansson P Petersson K Gustafasson S Qin J Olsson E Ilver D Krozer A Muhammed M Johansson C Tailored Magnetic Nanoparticles for Direct and Sensitive Detection of Biomolecules in Biological Samples Nano Lett 2008 8 3423 3428 10.1021/nl8022498 18754596

[B9] Kaittanis C Naser SA Perez JM One-Step, Nanoparticle-Mediated Bacterial Detection with Magnetic Relaxation Nano Lett 2007 7 380 383 10.1021/nl062553z 17298004

[B10] Wang D He J Rosenzweig N Rosenzweig Z Superparamagnetic Fe_2_O_3 _Beads-CdSe/ZnS Quantum Dots Core-Shell Nanocomposite Particles for Cell Separation Nano Lett 2004 4 409 413 10.1021/nl035010n

[B11] Jun YW Huh YM Choi JS Lee JH Song HT Kim S Yoon S Kim KS Shin JS Suh JS Cheon J Nanoscale Size Effect of Magnetic Nanocrystals and Their Utilization for Cancer Diagnosis via Magnetic Resonance Imaging J Am Chem Soc 2005 127 5732 5733 10.1021/ja0422155 15839639

[B12] Gao L-Z Wu J Lyle S Zehr K Cao L Gao D Magnetite Nano particle-Linked Immunosorbent Assay J Phys Chem C 2008 112 17357 17367 10.1021/jp805994h

[B13] Perez JM Simeone FJ Saeki Y Josephson L Weissleder R Virus-induced self assembly of magnetic nanoparticles allows the detection of viral particles in biological media J Am Chem Soc 2003 125: 10192 10193 10.1021/ja036409g 12926940

[B14] Qin J Laurent S Jo YS Roch R Mikhaylova M Bhujwalla ZM Muller RN Muhammed M A High-Performance Magnetic Resonance Imaging *T*_2 _Contrast Agent Adv Mater 2007 19 1874 1878 10.1002/adma.200602326

[B15] Yu MK Jeong YY Park J Park S Kim JW Min JJ Kim K Jon S Drug-Loaded Superparamagnetic Iron Oxide Nanoparticles for Combined Cancer Imaging and Therapy In Vivo Angew Chem Int Ed 2008 47 5362 5365 10.1002/anie.200800857 18551493

[B16] Nam JM Thaxton CS Mirkin CA Nanoparticle-Based Bio-Bar Codes for the Ultrasensitive Detection of Proteins Science 2003 301 1884 1886 10.1126/science.1088755 14512622

[B17] Sun S Zeng H Robinson DB Raoux S Rice PM Wang SX Li G Monodisperse MFe_2_O_4 _(M = Fe, Co, Mn) Nanoparticles J Am Chem Soc 2004 126 273 279 10.1021/ja0380852 14709092

[B18] Lee Y Lee J Bae CJ Park JG Noh HJ Park JH Hyeon T Large-Scale Synthesis of Uniform and Crystalline Magnetite Nanoparticles Using Reverse Micelles as Nanoreactors under Reflux Conditions Adv Funct Mater 2005 15 503 509 10.1002/adfm.200400187

[B19] Wang X Zhuang J Peng Q Li YD A general strategy for nanocrystal synthesis Nature 2005 437 121 124 10.1038/nature03968 16136139

[B20] Gao LZ Zhuang J Nie L Zhang JB Zhang Y Gu N Wang TH Feng J Yang DL Perrett S Yan X Intrinsic peroxidase-like activity of ferromagnetic nanoparticles Nat Nanotechnol 2007 2 577 583 10.1038/nnano.2007.260 18654371

[B21] Gupta AK Gupta M Synthesis and surface engineering of iron oxide nanoparticles for biomedical applications Biomaterials 2005 26 3995 4021 10.1016/j.biomaterials.2004.10.012 15626447

[B22] Thomas LA Dekker L Kallumadil M Southern P Wilson M Nair SP Pankhurst QA Parkin IP Carboxylic acid-stabilized iron oxide nanoparticles for use in magnetic hyperthermia J Mater Chem 2009 19 6529 6535 10.1039/b908187a

[B23] Kohler N Sun C Wang J Zhang M Methotrexate-Modified Superparamagnetic Nanoparticles and Their Intracellular Uptake into Human Cancer Cells Langmuir 2005 21 8858 8868 10.1021/la0503451 16142971

[B24] Fried T Shemer G Markovich G Ordered Two-Dimensional Arrays of Ferrite Nanoparticles Adv Mater 2001 13 1158 1161 10.1002/1521-4095(200108)13:15<1158::AID-ADMA1158>3.0.CO;2-6

[B25] Park J An K Hwang Y Park JG Noh HJ Kim JY Park JH Hwang NM Hyeon T Ultra-large-scale syntheses of monodisperse nanocrystals Nat Mater 2004 3 891 895 10.1038/nmat1251 15568032

[B26] Park SJ Kim S Lee S Khim ZG Char K Hyeon T Synthesis and Magnetic Studies of Uniform Iron Nanorods and Nanospheres J Am Chem Soc 2000 122 8581 8582 10.1021/ja001628c

[B27] Yinong Z Yi X Xiaowen L Douglas RK Agneta H Shivasankarappa G Magnus Hook Sthanam Narayana VL A'Collagen Hug' Model for Staphylococcus aureus CNA binding to collagen EMBO J 2005 24 4224 4236 10.1038/sj.emboj.7600888 16362049 PMC1356329

[B28] Darryl W N Katie Gold A Tracey PHR Sheryl EH Otto Wilson C Jr Surface modification of magnetic nanoparticles using gum Arabic J Nanopart Res 2006 8 749 753

[B29] Cullity BD Elements of X-ray Diffraction 1956 Addison-Wesley Publishing Company, INC

[B30] Zou G Xiong K Jiang C Li H Li T Du J Qian Y Fe_3_O_4 _Nanocrystals with Novel Fractal J Phys Chem B 2005 109 18356 18360 10.1021/jp052678c 16853363

[B31] Gao Y Chambers SA Heteroepitaxial growth of alpha-Fe_2_O_3_, gamma-Fe_2_O_3 _and Fe_3_O_4 _thin films by oxygen-plasma-assisted molecular beam epitaxy J Cryst Growth 1997 174 446 454 10.1016/S0022-0248(96)01141-4

[B32] Pileni MP The role of soft colloidal templates in controlling the size and shape of inorganic nanocrystals Nat Mater 2003 2 145 150 10.1038/nmat817 12612669

[B33] Shaswant BS Dong-Hwang C Fast removal of copper ions by gum Arabic modified nano-adsobent J Hazard Mater 2007 147 792 799 10.1016/j.jhazmat.2007.01.079 17321674

[B34] Mark HA Lisa BW Jonathan SD Rebecca R Sheila GA Influence of gold nanoparticles on collagen fibril morphology quantified using transmission electron microscopy and image analysis BMC Med Imaging 2006 6 4 10 10.1186/1471-2342-6-4 16737541 PMC1501007

[B35] Khaled NE Hanan IM Mona H Method for the Production of Bare (Non-functionalized) Multiple element Magnetic Nanoparticles and their use in Fast Detection and Removal of Pathogenic Bacteria from Water Resources Registered Patent (2450), Ministry of Industry and Trade, Amman, Jordan 2008

[B36] Speziale P Raucci G Visai L Switalski LM Timpl R Hook M Binding of Collagen to *Staphylococcus aureus *Cowan J of Bacteriology 1986 167 77 81 10.1128/jb.167.1.77-81.1986 PMC212843 3722129

